# Characterization of Diabetic Neuropathy in the Zucker Diabetic Sprague-Dawley Rat: A New Animal Model for Type 2 Diabetes

**DOI:** 10.1155/2014/714273

**Published:** 2014-10-13

**Authors:** Eric P. Davidson, Lawrence J. Coppey, Amey Holmes, Sergey Lupachyk, Brian L. Dake, Christine L. Oltman, Richard G. Peterson, Mark A. Yorek

**Affiliations:** ^1^Department of Internal Medicine, The University of Iowa, Iowa City, IA 52242, USA; ^2^Department of Veterans Affairs Iowa City Health Care System, Iowa City, IA 52246, USA; ^3^PreClinOmics Inc., Indianapolis, IN 46268, USA; ^4^Iowa City Veterans Administration Center for the Prevention and Treatment of Visual Loss, Iowa City, IA 52246, USA; ^5^Fraternal Order of Eagles Diabetes Research Center, University of Iowa, Iowa City, IA 52242, USA

## Abstract

Recently a new rat model for type 2 diabetes the Zucker diabetic Sprague-Dawley (ZDSD/Pco) was created. In this study we sought to characterize the development of diabetic neuropathy in ZDSD rats using age-matched Sprague-Dawley rats as a control. Rats were examined at 34 weeks of age 12 weeks after the onset of hyperglycemia in ZDSD rats. At this time ZDSD rats were severely insulin resistant with slowing of both motor and sensory nerve conduction velocities. ZDSD rats also had fatty livers, elevated serum free fatty acids, triglycerides, and cholesterol, and elevated sciatic nerve nitrotyrosine levels. The corneas of ZDSD rats exhibited a decrease in subbasal epithelial corneal nerves and sensitivity. ZDSD rats were hypoalgesic but intraepidermal nerve fibers in the skin of the hindpaw were normal compared to Sprague-Dawley rats. However, the number of Langerhans cells was decreased. Vascular reactivity of epineurial arterioles, blood vessels that provide circulation to the sciatic nerve, to acetylcholine and calcitonin gene-related peptide was impaired in ZDSD rats. These data indicate that ZDSD rats develop many of the neural complications associated with type 2 diabetes and are a good animal model for preclinical investigations of drug development for diabetic neuropathy.

## 1. Introduction

Animal models have been widely used for the study of the etiology of diabetic complications. One of the models that have been used for the study of type 2 diabetes has been the Zucker diabetic fatty (ZDF) rat [1–4]. My laboratory has shown that diabetic neuropathy develops with the onset of hyperglycemia in ZDF rats; slowing of motor nerve conduction velocity is preceded by impaired vascular relaxation of epineurial arterioles of the sciatic nerve [5, 6]. We have also demonstrated that diabetic vascular and neural complications in ZDF rats have a complex etiology and are difficult to reverse [6–8]. However, one concern for the applicability of the ZDF rats for the study of diabetic complications and translation to humans is the recessive homozygous mutation in the leptin receptor (*fa*) that causes loss of function and induces severe hyperphagia [9, 10]. Leptin has been shown to have an array of effects that may influence development of the metabolic syndrome [11–15]. Thus, loss of leptin signaling in the ZDF rat makes it a less than ideal model for complications of type 2 diabetes in humans who generally do not have a leptin receptor deficit. These concerns have led to the creation of the Zucker diabetic Sprague-Dawley (ZDSD) rat [9, 10]. The ZDSD rat was developed by cross-breeding the Charles River Laboratory diet-induced obese rats (Sprague-Dawley-derived) with lean ZDF^−/−^ rats. Selective inbreeding produced animals with a predisposition to obesity and a propensity to develop overt diabetes between 15 and 21 weeks of age with nutritional intervention [9]. Importantly, the ZDSD rats have an intact leptin signaling pathway and more modest accumulation of body fat in comparison to the ZDF rats [9]. The objective of this study was to characterize endpoints associated with diabetic neuropathy in ZDSD rats compared to age matched Sprague-Dawley rats.

## 2. Materials and Methods

Unless stated otherwise all chemicals used in these studies were obtained from Sigma Chemical Co. (St. Louis, MO).

### 2.1. Animals

Age matched male Sprague-Dawley (Harlan Laboratories, Indianapolis, IN) and ZDSD/Pco (provided by PreClinOmics, Indianapolis, IN) rats were housed in a certified animal care facility and food (Purina 5008, Richmond, IN) and water were provided ad libitum. All institutional (approval ACURF no. 1202032) and NIH guidelines for use of animals were followed. At each stage of the studies all possible means were taken to avoid animal suffering and pain. At 16 weeks of age both Sprague-Dawley and ZDSD rats were placed on Purina diet 5SCA for 6 weeks (nutrient content for the Purina 5008 and 5SCA diets were protein 23.5 versus 10.5%, fat 6.5 versus 25.6%, fiber 6.0 versus 3.8%, and carbohydrates 49.4 versus 50.6%). During this time 9 of the 12 ZDSD rats spontaneously developed hyperglycemia with nonfasting blood glucoses ranging from 387 to 558 mg/dL. The Sprague-Dawley rats maintained normal blood glucose levels during this period. Blood glucose levels were determined using glucose-oxidase reagent strips (Aviva Accu-Chek, Roche, Mannheim, Germany). After 6 weeks on the Purina 5SCA diet all rats were returned to the Purina 5008 diet and studied 12 weeks later. The three ZDSD rats that did not become hyperglycemic were excluded from the study.

### 2.2. Glucose Tolerance

Glucose tolerance was determined by injecting rats with a saline solution containing 2 g/kg glucose, i.p., after an overnight fast as previously described [16]. Rats were briefly anesthetized with isoflurane and the glucose solution was injected. Immediately prior to the glucose injection and at 15, 30, 45, 60, 90, 120, 180, and 240 min blood samples from the tip of the tail were taken to measure circulating glucose levels using glucose-oxidase reagent strips.

### 2.3. Thermal Nociceptive Response and Corneal Sensitivity

Thermal nociceptive response in the hindpaw was measured using the Hargreaves method as previously described [17]. Data was reported in sec. Corneal sensation was measured using a Cochet-Bonnet filament esthesiometer in unanaesthetized rats (Luneau Ophtalmologie, France) [18]. The testing began with the nylon filament extended to the maximal length (6 cm). The end of the nylon filament was touched to the cornea. If the rat blinked (positive response) the length of the filament was recorded. If the rat did not blink then the nylon filament was shortened by 0.5 cm and the test was repeated until a positive response was recorded. This process was repeated for each eye three times.

### 2.4. Motor and Sensory Nerve Conduction Velocity

On the day of terminal studies rats were weighed and anesthetized with Nembutal i.p. (50 mg/kg, i.p., Abbott Laboratories, North Chicago, IL). Motor and sensory nerve conduction velocities were determined as previously described using a noninvasive procedure in the sciatic-posterior tibial conducting system and digital nerve, respectively [17]. Motor and sensory nerve conduction velocities were reported in meters per second.

### 2.5. Corneal Innervation

Subbasal epithelial corneal nerves were imaged using the Rostock cornea module of the Heidelberg Retina Tomograph confocal microscope as previously described [18]. Briefly, the anesthetized rat was secured to a platform that allowed adjustment and positioning of the rat in three dimensions. A drop of GenTeal (lubricant eye gel) was applied onto the tip of the lens and advanced slowly forward until the gel contacted the cornea allowing optical but not physical contact between objective lens and corneal epithelium. Ten random high-quality images without overlap of the subbasal nerve plexus of the central cornea were acquired by finely focusing the objective lens to maximally resolve the nerve layer just under the corneal epithelium. For these studies a single parameter of corneal innervation was quantified [18]. Corneal nerve fiber length, defined as the total length of all nerve fibers and branches (in millimeters) present in the acquired image standardized for area of the image (in square millimeters), was determined for each image by tracing the length of each nerve in the image, summing the total length, and dividing by the image area [18]. The corneal fiber length for each animal was the mean value obtained from the ten acquired images and was expressed as mm/mm^2^. Based on receiver operating characteristic (ROC) curve analysis, corneal nerve fiber length is the optimal parameter for diagnosing patients with diabetic neuropathy and has the lowest coefficient of variation [19, 20].

### 2.6. Intraepidermal Nerve Fiber Density and Langerhans Cells in Skin from the Hindpaw

As previously described, immunoreactive nerve fiber profiles innervating the skin from the hindpaw and number of Langerhans cells were determined using standard confocal microscopy [16–18]. Samples of skin of the right hindpaw were fixed, dehydrated, and embedded in paraffin. Three sections (7 *μ*m) for each animal were collected and immunostained with anti-PGP9.5 antibody (rabbit anti-human, AbD Serotec, Morpho Sys US Inc., Raleigh, NC) overnight followed by treatment with secondary antibody Alexa Fluor 546 goat anti-rabbit. Immunostained nerve profiles and Langerhans cells were counted by two individual investigators that were masked to the sample identity. All immunoreactive profiles and cells were normalized to length [18].

### 2.7. Vascular Reactivity in Epineurial Arterioles

Video microscopy was used to investigate in vitro vasodilatory responsiveness of epineurial arterioles vascularizing the region of the sciatic nerve as previously described [21, 22]. The vessels used for these studies were generally oriented longitudinally in relation to the sciatic nerve; however, radially oriented vessels were also used on occasion. The arterioles used in this study should be regarded as epineurial rather than perineurial vessels. To isolate these vessels, the common iliac was exposed, and the branch points of the internal pudendal and superior gluteal arteries were identified. The vessels were then clamped, and tissue containing these vessels and branches at the internal pudendal and superior gluteal arteries were dissected en bloc. The block of tissue was immediately submerged in a cooled (4°C) and oxygenated (20% O_2_, 5% CO_2_, and 75% N_2_) Krebs-Henseleit physiological saline solution (PSS) of the following composition (in millimoles per liter): NaCl 118, KCl 4.7, CaCl_2_ 2.5, KH_2_PO_4_ 1.2, MgSO_4_ 1.2, NaHCO_3_ 20, Na_2_EDTA 0.026, and glucose 5.5. Branches of the superior gluteal and internal pudendal arteries (50 to 150 *μ*m internal diameter and 1-2 mm in length) were carefully dissected and trimmed of fat and connective tissue. Both ends of the isolated vessel segment were cannulated with glass micropipettes filled with PSS (4°C) and secured with 10–0 nylon Ethilon monofilament sutures (Ethicon, Cornelia, GA). The pipettes were attached to a single pressure reservoir (initially set at 0 mmHg) under condition of no flow. The organ chamber containing the cannulated vessels was then transferred to the stage of an inverted microscope (CK2; Olympus, Lake Success, NY). Attached to the microscope were a closed-circuit television camera (WV-BL200; Panasonic, Secaucus, NJ), a video monitor (Panasonic), and a video caliper (VIA-100K; Boeckeler Instruments, Tucson, AZ). The organ chamber was connected to a rotary pump (Masterflex; Cole Parmer Instrument, Vernon Hills, IL), which continuously circulated 37°C oxygenated PSS at 30 mL/min. The pressure within the vessel was then slowly increased to 40 mmHg. At this pressure, we found that KCl gave the maximal constrictor response. Therefore, all of the studies were conducted at 40 mmHg. Internal vessel diameter (resolution of 2 *μ*m) was measured by manually adjusting the video micrometer. After a 30 min equilibration, KCl was added to the bath to test vessel viability. Vessels failing to constrict by at least 30% were discarded. After they were washed with PSS, vessels were incubated for 30 min in PSS and then constricted with U46619 (10^−8^ to 10^−7^ mol/l) (Cayman Chemical, Ann Arbor, MI) to 30–50% of passive diameter. Afterwards, cumulative concentration-response relationships were evaluated for acetylcholine (10^−8^–10^−4^ M) and calcitonin gene-related peptide (10^−11^–10^−8^ M). At the end of each dose response curve for acetylcholine or calcitonin gene-related peptide papaverine (10^−5^ M) was added to determine maximal vasodilation.

### 2.8. Biological and Oxidative Stress Markers

Additional measurements were taken including nonfasting blood glucose and hemoglobin A_1_C levels (Glyco-Tek affinity column, Helena Laboratories, Beaumont, TX). Serum was collected for determining levels of free fatty acid, triglyceride, free cholesterol, and leptin, using commercial kits from Roche Diagnostics, Mannheim, Germany; Sigma Chemical Co., St. Louis, MO; Bio Vision, Mountain View, CA; and ALPCO diagnostics, respectively. Liver segments were collected for analysis of triglyceride deposition. Liver samples were embedded in Tissue-Tek O.C.T. compound (Sakura Finetek, Torrance, CA), sectioned (10 *μ*m thickness), and stained with oil red O to determine area of triglyceride deposition in each group by NIH Image [23]. Segments of sciatic nerve were used to determine nitrotyrosine staining as a marker of oxidative stress by Western blot analysis as previously described [24].

### 2.9. Data Analysis

The results are presented as mean ± S.E.M. Comparison between Sprague-Dawley and ZDSD rats was conducted using Student's *t*-test (Prism software; GraphPad, San Diego, CA). Concentration response curves for acetylcholine and calcitonin gene-related peptide were compared using a two-way repeated measures analysis of variance with autoregressive covariance structure using proc mixed program of SAS [21]. A *P* value of less than 0.05 was considered significant.

## 3. Results and Discussion

Data in Table 1 show that at 8 weeks of age ZDSD rats weighed significantly more than Sprague-Dawley rats. However, at 34 weeks of age Sprague-Dawley rats weighed significantly more than the ZDSD rats. Prior to becoming hyperglycemic ZDSD rats are modestly obese compared to Sprague-Dawley rats but with the onset of hyperglycemia weight gain is reduced. At the end of the study period ZDSD rats had a significantly higher nonfasting blood glucose level and elevated hemoglobin A_1_C value compared to Sprague-Dawley rats. Compared to Sprague-Dawley rats, at 34 weeks of age, ZDSD rats were hyperlipidemic as indicated by significantly increased serum free fatty acid, triglyceride, and cholesterol levels. Liver lipid levels were also significantly increased as indicated by significantly higher oil red staining of liver from ZDSD rats compared to Sprague-Dawley rats. Data in Table 1 also demonstrate that, in the sciatic nerve nitrotyrosine levels, a marker of oxidative stress is significantly increased.

**Figure 1:**
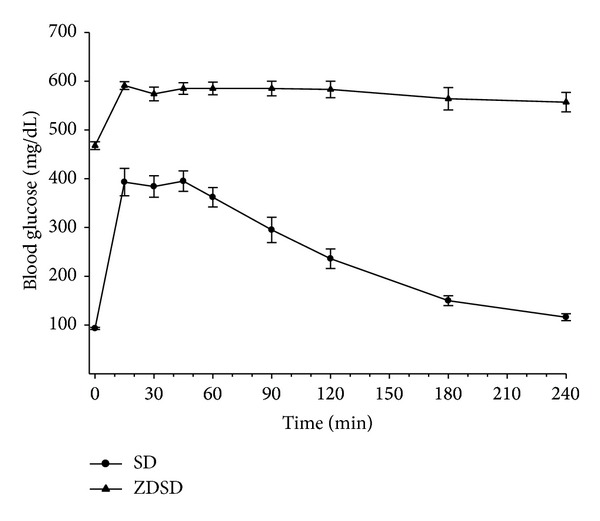
Glucose tolerance in age-matched Sprague Dawley and ZDSD rats. Glucose clearance was determined as described in the Methods section in Sprague Dawley (SD) and ZDSD rats. Data are presented as the mean ± S.E.M. for glucose utilization in mg/dL. The number of rats in each group was 10 and 9, respectively.

Data in Figure 1 demonstrate that at 34 weeks of age, following a minimum of 12 weeks of hyperglycemia, ZDSD rats are severely glucose intolerant and insulin resistant as indicated by a very slow rate of glucose clearance compared to Sprague-Dawley rats.

**Figure 2:**
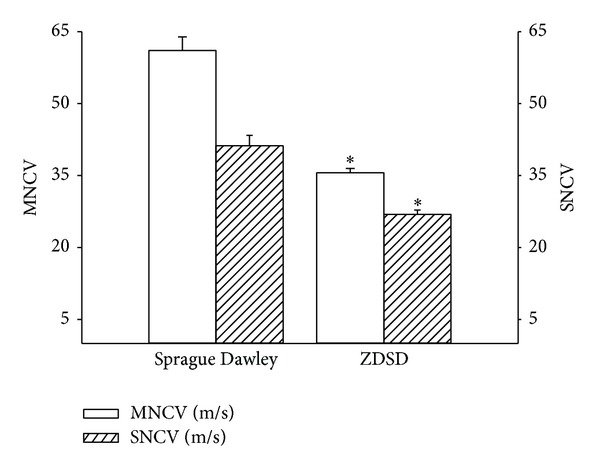
Motor and sensory nerve conduction velocity in age-matched Sprague Dawley and ZDSD rats. Motor and sensory nerve conduction velocities were examined as described in Section 2. The number of rats in each group was 10 and 9, respectively. Data are presented as the mean ± S.E.M for motor and sensory nerve conduction velocities in m/sec. *P < 0.05 compared to Sprague Dawley rats.

**Figure 3:**
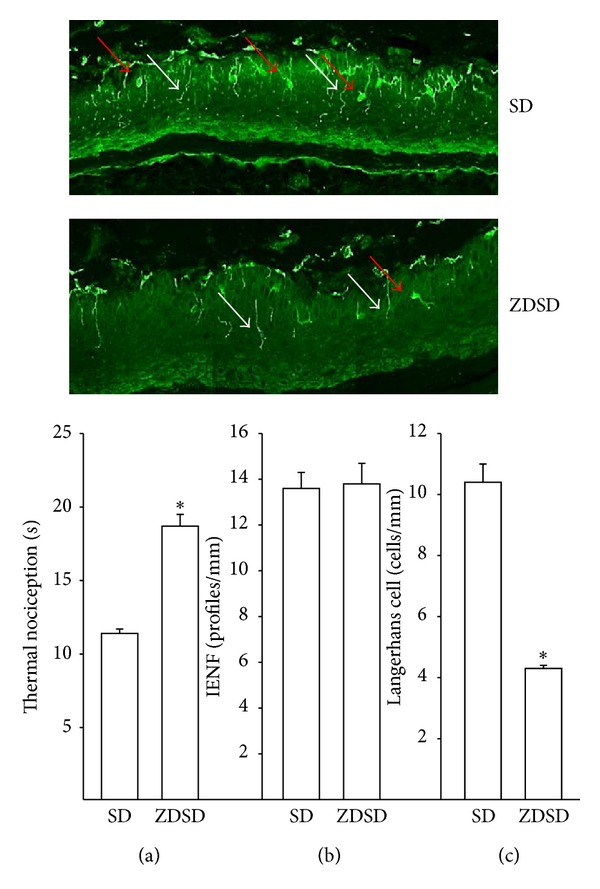
Thermal nociception, intraepidermal nerve fiber, and Langerhans cell density in age-matched Sprague Dawley and ZDSD rats. Thermal nociception (a), intraepidermal nerve fiber (b), and Langerhans cell (c) density were examined as described in the Methods section in Sprague Dawley (SD) and ZDSD rats. Inserts are representative images of skin from foot pads from Sprague-Dawley (left) and ZDSD (right) rats. The white arrows point out nerve fibers and the red arrows point out the Langerhans cell. The number of rats in each group was 10 and 9, respectively. Data are presented as the mean ± S.E.M. for thermal nociception in sec, intraepidermal nerve fiber as profiles per mm, and Langerhans cell as number of cells per mm. *P < 0.05 compared to Sprague Dawley rats.

A number of neural endpoints relating to diabetic neuropathy were examined in order to verify the extent of nerve damage that occurs in ZDSD rats. Determination of nerve conduction velocity is a standard endpoint used to study the effect of diabetes on nerve function. Data in Figure 2 demonstrate that both motor and sensory nerve conduction velocities are significantly slower in ZDSD rats compared to Sprague-Dawley rats at 34 weeks of age. More recently determining changes in small sensory nerve structure and function in the skin and cornea have been proposed as surrogate markers for diabetic neuropathy [19]. Data in Figure 3(a) demonstrate that ZDSD rats are thermal hypoalgesic as indicated by longer latency of withdrawal of their foot from a thermal stimulus compared to Sprague-Dawley rats. However, unlike other diabetic rodent models, the onset of chronic hyperglycemia did not cause a decrease in intraepidermal nerve fibers in the skin from the hindpaw (Figure 3(b)). In contrast, there was a significant decrease in the number of Langerhans cells (Figure 3(c)). These data suggest that impairment of thermal sensitivity in diabetes is not always associated with a decrease in intraepidermal nerve fiber density. This has previously been reported by others [25]. Beiswenger et al. reported that streptozotocin-diabetic mice developed thermal hypoalgesia prior to a measurable reduction in immunoreactive nerve fibers [25]. The decrease in Langerhans cells in ZDSD rats, after a minimum of 12 weeks of hyperglycemia, contrasts with what has been reported in streptozotocin-diabetic Sprague-Dawley rats [26]. Lauria et al. reported a reduction in intraepidermal nerve fiber density and a significantly higher density of Langerhans cells in the skin of the hindpaw [26]. In contrast, it has been reported that Langerhans cell density is significantly decreased in recently diagnosed type 2 diabetes patients [27]. In the latter study it was suggested that loss of Langerhans cells could promote a cutaneous immunogenic imbalance toward inflammation predisposing to polyneuropathy and foot ulcers [27]. Additional studies are needed to determine the role a change in the density of Langerhans cells may have in the development of diabetic neuropathy.

**Figure 4:**
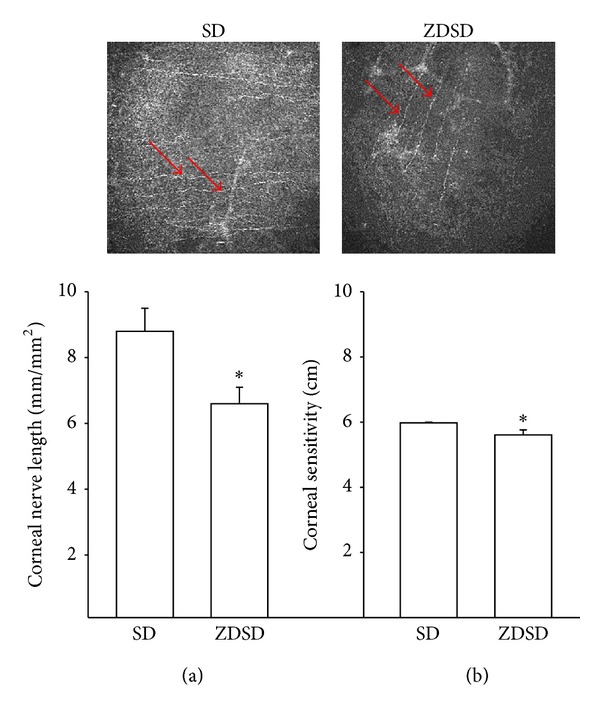
Corneal nerve fiber length and corneal sensitivity in age-matched Sprague Dawley and ZDSD rats. Corneal nerve fiber length (a) and corneal sensitivity (b) were examined as described in the Methods section in Sprague Dawley (SD) and ZDSD rats. Inserts are representative images of the subepithelial layer of the cornea from Sprague-Dawley (left) and ZDSD (right) rats. The red arrows point out the corneal nerves. The number of rats in each group was 10 and 9, respectively. Data are presented as the mean ± S.E.M. for corneal nerve fiber length in mm/mm^2^ and corneal sensitivity in cm. *P < 0.05 compared to Sprague Dawley rats.

Data in Figure 4 demonstrate that nerves in the subepithelial layer of the cornea (a) are decreased and corneal sensitivity (b) is impaired in ZDSD rats compared to Sprague-Dawley rats. Even though chronic hyperglycemia does not appear to cause a decrease in intraepidermal nerve fiber density in the skin we did observe a significant decrease in the overall length of nerves in the subbasal epithelial layer of the cornea. This suggests that the pathophysiology controlling nerve fiber density in the skin and cornea may be different. Ziegler et al. recently reported in recently diagnosed type 2 diabetic patients that corneal confocal microscopy and skin biopsy both detected nerve fiber loss but largely in different patients [28], creating what they term as a patchy manifestation pattern of small fiber neuropathy in recently diagnosed type 2 diabetes [28].

The lack of a decrease in intraepidermal nerve fibers in the skin of the hindpaw of ZDSD rats along with thermal hypoalgesia was surprising. Decreased intraepidermal nerve fiber density has been widely reported to occur in rodent models of type 1 and type 2 diabetes including Zucker diabetic fatty rats and in humans with diabetes [29, 30]. We reported a correlation between decreased intraepidermal nerve fiber density and corneal nerve fiber density in type 2 diabetic rats [31]. It is not known whether the intraepidermal nerve fibers in ZDSD rats remain functionally normal. It is possible that the neuropeptide content of the intraepidermal nerve fibers in ZDSD rats is impaired. This could explain the decrease in thermal nociception in ZDSD rats even though the number of nerve fibers present in the skin is normal. Additional studies are needed to address this paradox.

**Figure 5:**
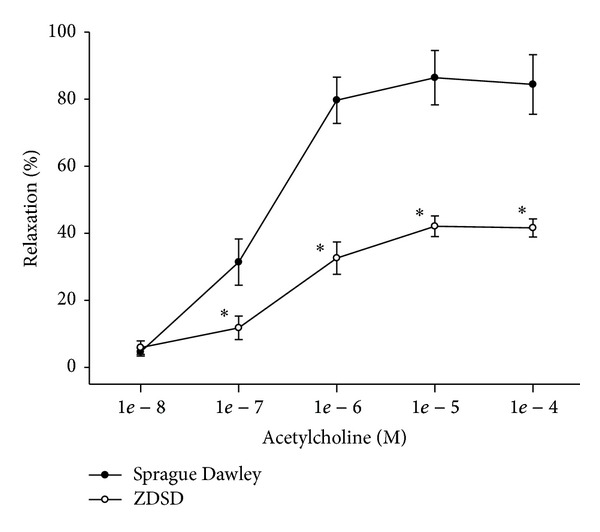
Vascular relaxation by acetylcholine in epineurial arterioles from age-matched Sprague Dawley and ZDSD rats. Pressurized arterioles (40 mm Hg and ranging from 60 to 100 *μ*m luminal diameter) were constricted with U46619 (30–50%) and incremental doses of acetylcholine were added to the bathing solution while recording steady state vessel diameter. Data are presented as the mean of % relaxation ± S.E.M. The number of rats in each group was 10 and 9 for Sprague Dawley and ZDSD, respectively. *P < 0.05 compared to Sprague Dawley rats.

**Figure 6:**
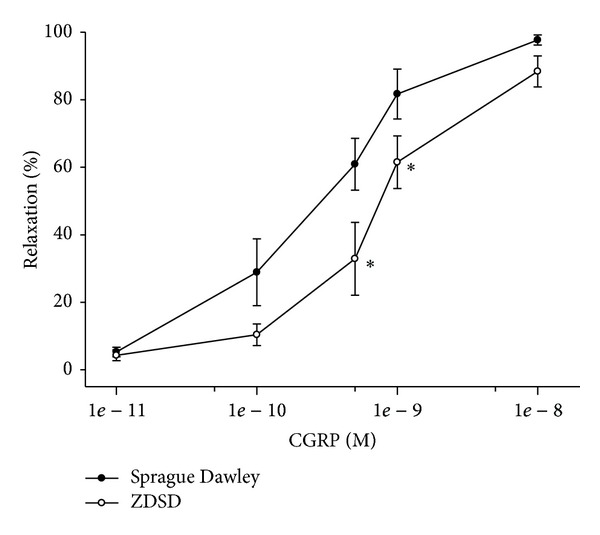
Vascular relaxation by calcitonin gene-related peptide in epineurial arterioles from age-matched Sprague Dawley and ZDSD rats. Arterioles were treated as described in Figure 5. Incremental doses of calcitonin gene-related peptide (CGRP) were added to the bathing solution while recording steady state vessel diameter. Data are presented as the mean of % relaxation ± S.E.M. The number of rats in each group was 10 and 9 for Sprague Dawley and ZDSD, respectively. *P < 0.05 compared to Sprague Dawley rats.

We have previously demonstrated that diabetes causes a significant decrease in vascular relaxation of epineurial arterioles in type 1 and type 2 diabetic rat models [5–7, 17, 21, 22, 26, 32, 33]. We have theorized that reduced vascular reactivity of these blood vessels that supply circulation to the sciatic nerve could be a contributing factor to the development of diabetic neuropathy since vascular impairment precedes slowing of nerve conduction velocity [6, 21]. In these studies we demonstrate that vascular relaxation of epineurial arterioles from ZDSD rats to acetylcholine (Figure 5) and calcitonin gene-related peptide (Figure 6) is significantly impaired compared to Sprague-Dawley rats. Vascular relaxation to acetylcholine by small coronary and mesenteric arteries was also examined using previously described methodology and was found to be impaired (data not shown) [34, 35].

## 4. Conclusions

Chronic hyperglycemia in ZDSD rats causes vascular and neural dysfunction that is similar to what we have documented in other diabetic rat models and consistent with the development of diabetic neuropathy [5, 6, 16, 21, 22, 26]. The one exception is that chronic hyperglycemia in ZDSD rat did not cause a decrease in intraepidermal nerve fibers in the skin as has been reported for other diabetic rat models but there was a decrease in Langerhans cells. Overall, the ZDSD rat is an easily maintained animal model for type 2 diabetes that spontaneously develops hyperglycemia with dietary manipulation. This is followed by the development of diabetic neuropathy including loss of nerves in the subbasal epithelial layer of the cornea and decrease in corneal function. Since determination of cornea nerve density and function are noninvasive and surrogate markers for diabetic neuropathy, the ZDSD rat with its functional leptin pathway may be a good model for preclinical testing of treatments for diabetic neuropathy [19].

## Figures and Tables

**Table 1:** Vital parameters for Sprague Dawley and ZDSD rats.

Determination	Sprague Dawley (10)	ZDSD (9)
Start weight (g)	279 ± 3	300 ± 5^a^
End weight (g)	527 ± 11	424 ± 15^a^
Blood glucose (mg/dL)	140 ± 5	564 ± 11^a^
Hb A_1_C (%)	7.0 ± 0.3	15.6 ± 0.6^a^
Serum free fatty acid (mmol/L)	0.14 ± 0.02	0.52 ± 0.07^a^
Serum triglycerides (mg/dL)	104 ± 16	618 ± 129^a^
Serum free cholesterol (mg/mL)	1.7 ± 0.2	4.4 ± 0.7^a^
Liver oil red staining (% of total area)	2.2 ± 0.2	5.3 ± 0.6^a^
Sciatic nerve nitrotyrosine (arbitrary light units)	100 ± 11	212 ± 28^a^

For these studies there were 2 groups: Sprague Dawley (control) and ZDSD rats. Data are presented as the mean ± S.E.M. ^a^ *P* < 0.05 compared to control. The number of animals in each group is shown in parenthesis.
